# Effect of ultrasonic vibration on the mechanical properties of 3D printed acrylonitrile butadiene styrene and polylactic acid samples

**DOI:** 10.1016/j.heliyon.2023.e17053

**Published:** 2023-06-07

**Authors:** Shajahan Maidin, Thavinnesh Kumar Rajendran, Norilani Md Nor Hayati, Yap Yeong Sheng, Shafinaz Ismail, Ahmad Hilmi Muhammad

**Affiliations:** a Faculty of Manufacturing Engineering, Universiti Teknikal Malaysia Melaka, 76100, Melaka, Malaysia; b Pebble3D Sdn Bhd, Selangor, Malaysia

**Keywords:** Ultrasonic vibration, Material extrusion, Process parameters, Surface roughness, Microstructure analysis, Compressive strength

## Abstract

Fused deposition modeling (FDM) is an extrusion-based AM process that is widely used due to its cost-effectiveness and user friendly. However, FDM also has some limitations such as the appearance of seam lines between layers and the production of excess material residue leading to poor surface finish, poor bonding between layers and porosity. This paper presents the findings on the application of ultrasonic vibration in an open-source FDM 3D printer to investigate its effect on the mechanical properties and microstructure of acrylonitrile butadiene styrene (ABS) and Polylactic Acid (PLA) samples. Two units of ultrasonic piezoelectric transducer were clamped horizontally on the surface of the 3D printer platform. The ultrasonic vibration was transmitted directly to the platform while the sample received vibration with a specific frequency while the printing process commences. Two process parameters, namely build orientation and ultrasonic vibration were selected to analyze their significance and optimization on the mechanical properties and the microstructure of the printed samples. High compressive and low surface roughness are required to have the best properties for the printed sample. Therefore, the optimization parameters are performed with these settings where the compressive strength is maximized and the surface roughness is minimized. The result shows that the overall compressive strength in ABS and PLA samples created in the Z-axis orientation is higher than in the X-axis orientation. However, the compressive strength of ABS and PLA samples is not much different after the ultrasonic vibration was applied during the printing process. The microstructure analysis shows that bonding between the layers is similar when applying ultrasonic vibration for both ABS and PLA samples. Furthermore, the result indicates that the surface roughness increased at 10 kHz and then decreased or became smoother at 20 kHz for both ABS and PLA material samples. The analysis shows that the build orientation significantly affects the compressive strength in ABS and PLA samples. However, the ultrasonic vibration has no considerable impact. In surface roughness, the build orientation and ultrasonic vibration significantly affect ABS samples. However, the PLA samples are only slightly affected. The optimum parameters for both materials are found where Z-axis orientation and 0 kHz of the ultrasonic vibration samples gave the best compressive strength and surface roughness value.

## 1 Introduction

Additive manufacturing (AM) is a popular technology currently. AM has been applied in many fields, such as automotive, aerospace, engineering, medicine, biological system, and the food industry. AM is about a model created by using a three-dimensional Computer-Aided Design (CAD) system and fabricated without the aid of any tooling as in the conventional manufacturing process [1]. The parts will be made in numerous thin cross-sections of layers bonded together to approximate sizes similar to the CAD drawing data. AM technologies brought many advantages, such as feasibility in creating a complex design, cost-reducing, lead time reduction, and high repeatability [2]. Various advantages can be achieved using AM techniques, especially in design and manufacturing. In AM process, tooling is not required and the development of products can be achieved quickly [3]. Therefore, the user can customize and refine the product in a shorter time and lower cost. Furthermore, the on-demand production can be enhanced as the product can be printed only once.

Moreover, AM techniques can reduce the waste material of output compared to traditional manufacturing techniques, leading to lesser energy used. The manufacturing industries have evolved AM techniques due to the rapidity, flexibility, and cost-efficiency of AM techniques in design and manufacturing applied in different sectors [4]. However, compared with the traditional manufacturing process, AM mass production is hard to achieve due to the slow printing process and material limitations [5]. Furthermore, the cost per product may be high due to the high price of the material used and the absence of economic scale. Lastly, the poor surface quality caused by the staircase effect in the printing process had to be solved using post-processing techniques, leading to increased costs [6].

Fused Deposition Modeling (FDM) is an extrusion-based AM process that manufactures parts through a heated nozzle or orifice. FDM process is more popular than other AM processes due to the various advantages, such as ease to use and additional equipment not being required [7]. These advantages also brought other benefits like the reduced cost for the machine and processes due to the ease of use and extra machining tools that are unneeded. Furthermore, the thermoplastics material used in the FDM process had good chemical properties like low toxicity, safety, and ease of handling. Moreover, the characteristics of thermoplastic material provide a sustainable cycle as the product fabricated is recyclable. Due to the FDM technology being easy to use, cost-effective, and environmentally friendly, the actual industry widely uses it to create the prototype of products [8].

Although the FDM process has many advantages and has been developed for over 20 years, it still has its limitations in several parts. FDM process had a low printing speed, leading to a low production rate compared to other AM processes [9]. In addition, poor surface quality was a significant limitation for FDM printed parts, and post-processing is required to improve the surface roughness [10]. Besides surface roughness, the FDM printed parts still have various defects such as anisotropy, dimensional accuracy, and dependence on processes used, which were the problems faced in FDM technology [11]. Furthermore, repeated heating, and cooling cycles during the printing process, result in inconsistent mechanical properties due to residual stress that develops from the layer-by-layer printing process [[12], [13], [14], [15]]. Due to high residual stress, the strength of the printed part decreases, which leads to crack formation and warpage [[16], [17], [18]]. In addition, the seam line appears between layers of printed parts, and excess material is often left as a residue [[19], [20], [21], [22]]. Lastly, FDM process is currently only appropriate for small to medium-scale production with complex and non-safety essential components due to the lack of consistency in part and material quality [23]. In the FDM process, thermoplastic filaments such as PLA and ABS are commonly used as the printing material to create the parts [24].

Most of the published information related to AM parts surface finish improvement concentrated on the post-processing activities such as heat or chemical treatment, barrel or hand finishing, spray painting, CNC machining or linishing [[25], [26], [27], [28], [29]]. However, these post-process methods require additional cost, time, labour and hazardous. During the printing process, strategies such as build orientation optimization, slicing strategy optimization and process parameter optimization were also introduced [[30], [31], [32]]. However, after the printing process is completed, it still requires additional post-process methods stated above.

In this era of globalization, ultrasonic techniques have been developed and applied in various sectors of the industry [33]. Ultrasonic additive manufacturing (UAM) is a solid-state 3D printing technology that uses ultrasonic vibration to manufacture the parts through the layer-by-layer process [34]. Using the UAM process, an intelligent metal structure can be fabricated. The UAM process can create a metal matrix with different components by using a high degree of plastic metal flow and relatively low temperatures in the layer bonding process [35]. In our previous study, ultrasonic technology proved that the surface roughness of FDM printed parts is enhanced due to the reduction in layer thickness [36].

Nonetheless, Taguchi L9-based orthogonal array is used to evaluate the effect of process parameters on the mechanical properties of FDM printed parts. The Taguchi method is a statistical method and design of experiment (DOE) developed by Genichi Taguchi to improve the quality of products commonly applied to the engineering field. The difference between the Taguchi method and the conventional DOE method is the Taguchi method is dependent on decreases in the variation of data. In contrast, the traditional method of DOE is based on the specification and mean of data [37]. Furthermore, Taguchi has suggested an orthogonal array concept, which uses a fractional factorial design with unique orthogonal arrays where rows are represented as treatment combinations [38]. In contrast, the column heading described the factors. It is a helpful tool for estimating the significant factor affecting the result's performance [39].

Wu et al., (2018) have used ultrasonic vibration to strengthen ABS samples after the sample was printed, which is a process of compacting the ABS sample [40]. In addition, there is also an article that uses ultrasonic vibration on the printer's nozzle and provides data only for ABS microstructure [41]. There is no data on mechanical properties provided. However, the current article thoroughly investigates the ultrasonic tranducer's application at the printer platform for a constant and smooth vibration while the printing commences. A piezoelectric transducer will configure the frequency of vibration at different levels during the printing process of samples. Various process parameters such as build orientation, layer thickness, raster angle and infill pattern, are chosen to be investigated as the significant factors affecting FDM printed part’s compressive strength and surface roughness. The optimization was performed using the Taguchi method, showing that optimizing the process parameters using Taguchi L9 orthogonal array is successful. The ultrasonic-assisted FDM printed samples were compared with the standard printed parts to analyze the improvement in the mechanical properties and mircostructure.

## 2 Methodology

### 2.1 Design of experiment

In this experiment, Taguchi L6 orthogonal array was used to design the experiment based on the process parameters selected: vibration frequency and build orientation. Table 1 shows the process parameters that were used in the experiment where vibration frequency is in three levels (0 kHz, 10 kHz, and 20 kHz), the layer thickness is fixed at 0.2 mm, the raster angle is fixed at 30°, build orientation is in two directions (X and Z), and infill pattern 100% is fixed with grid pattern. Table 2 shows the setting of six trials designed with Taguchi L6 orthogonal array to identify the significant and optimum parameters for build orientation and ultrasonic frequency.

**Table 1:** The control factors for compressive strength and surface roughness.

Control Factors	Unit	Levels
	1 2	3
Build Orientation	X Z	–
Ultrasonic Frequency	(kHz) 0 10	20
Layer thickness	(mm) 0.2 -	–
Raster Angle	(^*◦*^) 30 -	–
Infill Pattern	Grid -	–

**Table 2:** Experimental data gathering plan derived based on L_6_ Taguchi Method.

$L_{6}$ (32) Orthogonal Array				
No. of Run	Control Factors		Response Value	
	Build Orientation	Ultrasonic Frequency (kHz)	Compressive Strength (MPa)	Surface Roughness, Ra (μ)
1	X	0	Q1	P1
2	X	10	Q2	P2
3	X	20	Q3	P3
4	Z	0	Q4	P4
5	Z	10	Q5	P5
6	Z	20	Q6	P6

### 2.2 Analysis of variance (ANOVA)

In the experiment, the analysis of variance (ANOVA) was performed to identify the significant influence of process parameters on the mechanical properties of samples. It is used due to its ability to prove that the factors are statistically significant if the P-value for the combination of factors is lower than 0.05. Then a regression equation will be generated in the actual unit or coded. The model will then be validated with a model diagnostic report. Finally, the optimization of the parameters will be conducted to identify the best combination of parameters used in the ultrasonic-assisted printing process by setting the target value of compressive strength at maximum and surface roughness at minimum. For this experiment, ANOVA was performed using Minitab17 software.

### 2.3 Experimental set-up

**Fig. 1:**
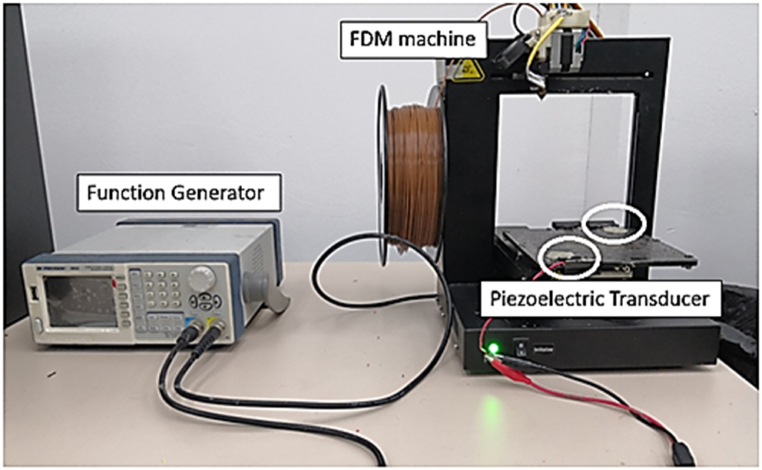
Experimental set-up of ultrasonic-assisted FDM system.

As illustrated in Fig. 1, there are four main components required for the ultrasonic-assisted FDM system: a laptop with 3D printing software, FDM printer, an ultrasonic piezoelectric transducer, and a function generator. The ultrasonic piezoelectric transducer was clamped horizontally on the surface of the 3D printer platform and the vibration was transmitted thoroughly on the printer platform while the printing process commences. The ultrasonic piezoelectric transducer was not attached to the 3D printer nozzle due to its high temperature from the heated block and this could damage the ultrasonic piezoelectric transducer. The ultrasonic piezoelectric transducer was attached to the build platform of the FDM machine and connected with a function generator to adjust the vibration frequency of the piezoelectric transducer. Two units of the ultrasonic piezoelectric transducer were used in the experiment. Plasticine was used to fix the ultrasonic piezoelectric transducer onto the printer platform so that the vibration is transmitted without losing the vibration or signal and to avoid damaging the ultrasonic piezoelectric transducer.

**Fig. 2:**
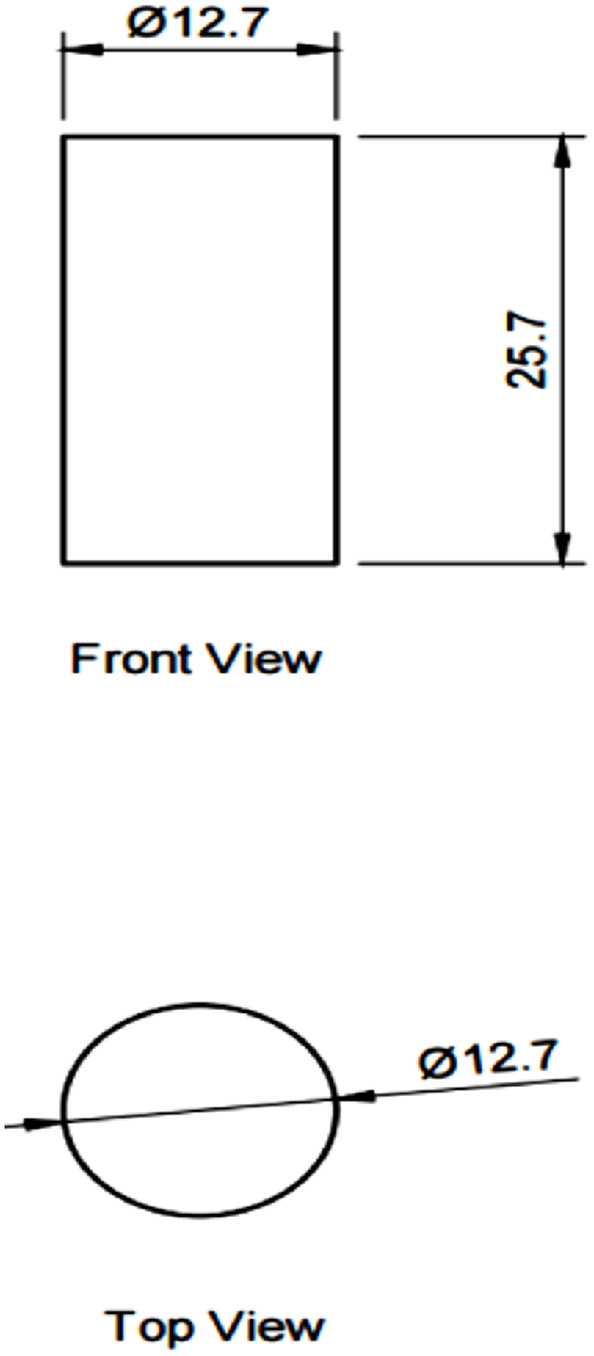
CAD model of the compressive test sample.

**Fig. 3:**
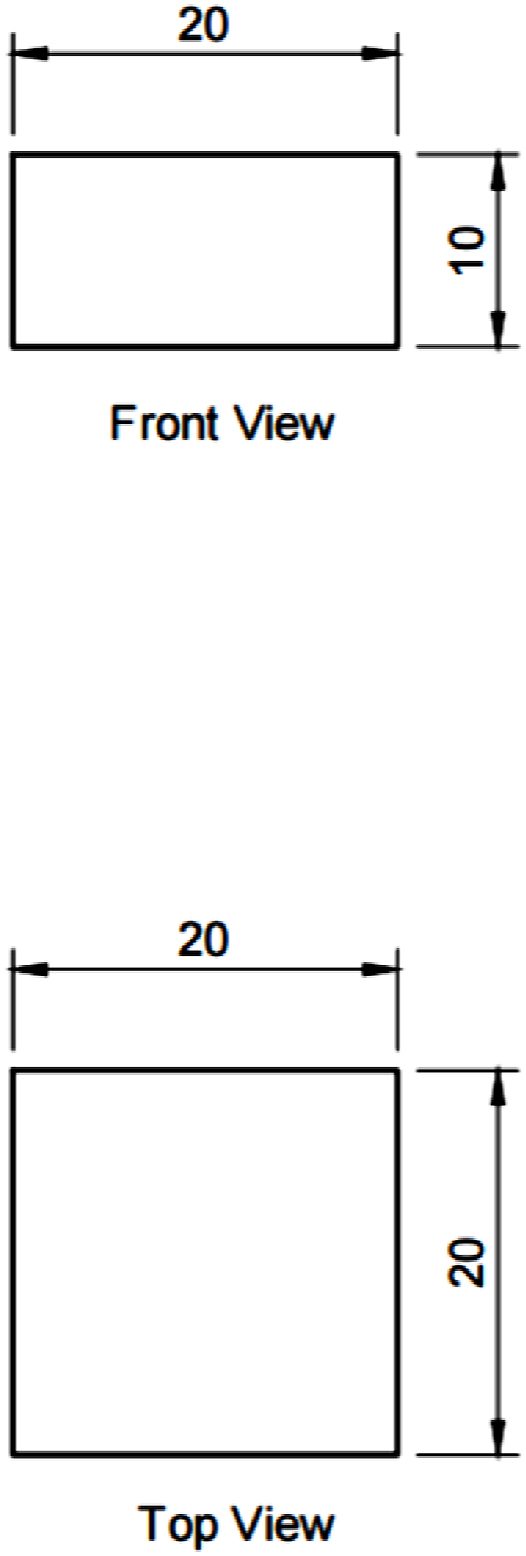
CAD model of the surface roughness sample.

The printing software is used to adjust the printing parameters of the FDM printer to ensure the samples are printed according to the settings. Once all the components are ready, the printing process is started until all the samples are printed. Then, the printed samples were sent for mechanical testing, and the data collected were analyzed through ANOVA to identify the significant and optimum parameters. The data obtained was analyzed, discussed, and compared with the effect of process parameters and their difference on ABS and PLA materials. In this experiment, 48 (6 × 3 × 2 + 6 × 2) samples were printed to identify significant and optimum parameters due to the two different types of design for samples (surface roughness and compressive strength) and two types of material used: ABS and PLA. The samples were designed for the compressive test into a cylinder size of 12.7 mm × 25.4 mm, according to ASTM D695-15, as shown in Fig. 2, while for surface roughness, the samples were designed into a square shape with 20 mm × 20 mm x 10 mm, as shown in Fig. 3.

**Fig. 4:**
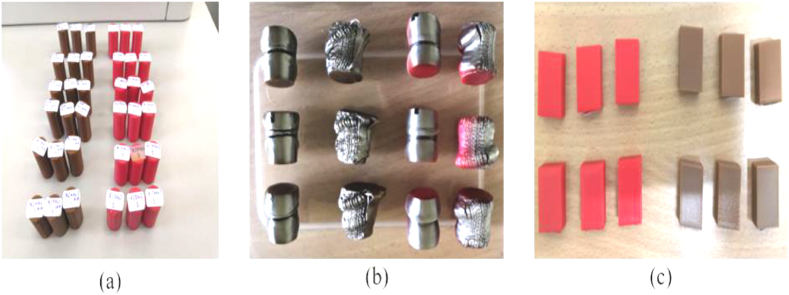
ABS (red) and PLA (brown) samples that were used for (a) compressive test, (b) microstructure inspection, and (c) surface roughness

Fig. 4 shows the samples printed in red for the ABS material and brown for the PLA material. Fig. 4(a) shows samples for compressive tes. Fig. 4(b) shows samples for microstructure inspection. Fig. 4 (c) shows samples for surface roughness. For the compressive and surface roughness testing, each experiment was conducted three times to ensure the accuracy of the data obtained. For the microstructure inspection, the samples from the compressive test were used. Mitutoyo Suftest SJ-301 was used to evaluate the surface roughness of the samples. The test was performed through a probe placed on the last surface area of samples and was measured according to ISO 1997 standard. The test was performed with an evaluation length of 2.4 mm and repeated 3 times.

Furthermore, the Shimadzu Autograph AGS-X 20 kN Universal Testing Machine was used to perform the compression test. In the compressive test, a 250 kN fixed-type compression plate jig was used to evaluate the compressive properties of the samples. The compression plate presses the samples at 1.2 mm/min at uniform speed until the compression plate reaches half of the height for the samples. As ABS and PLA are polymers with poor conductivity, the sputter coating process was required for the samples before the inspection by using oxidizing metals, including platinum, palladium, gold, and silver. The coating process was performed through a sputter coater by adjusting the chamber pressure to 13.9 mbar and the plasma process current to 30 mA. After the coating process, the microstructure of standard and ultrasonic-assisted printed samples was observed using Scanning Electron Machine with 15 keV acceleration voltage and 20, 50, 100 and 300 times magnification to compare and analyze their difference.

## 3 Result and discussion

### 3.1 Result of compressive strength and surface roughness

Table 3 shows the average compressive strength and surface roughness results of ABS samples. Based on the result, the samples built with Z-axis orientation showed higher compressive strength than those built with X-axis orientation. The highest value in X-axis orientation is 28.8274 MPa at 0 kHz, and Z orientation is 36.8588 MPa at 10 kHz. In X-axis orientation, the compressive strength decreases when the ultrasonic vibration frequency increases. However, in Z orientation, the compressive strength increased by about 0.0109% at 10 kHz while decreasing by about 1.7843% at 20 kHz compared to 0 kHz. As the result of surface roughness for ABS samples, the samples built with X-axis orientation have a lower value than the Z orientation. When the ultrasonic vibration frequency increases, the surface roughness of the samples built with X orientation increases by about 12.9432% at 10 kHz and 5.7285% at 20 kHz.

**Table 3:** Result of compressive strength and surface roughness for ABS printed samples and its improvement using ultrasonic-assisted FDM.

Run	Control Factors		Response Values		Improvement compared to 0 kHz (%)	
	Build Orientation	Ultrasonic Frequency (kHz)	Compressive Strength (MPa)	Surface Roughness, Ra (μm)	Compressive Strength (MPa)	Surface Roughness, Ra (μm)
1	X	0	28.8274	7.6233	–	–
2	X	10	28.2819	8.61	−1.8923	12.94321
3	X	20	27.6912	8.06	−3.9414	5.72849
4	Z	0	36.8548	8.6567	–	–
5	Z	10	36.8588	20.92	0.0109	141.6625
6	Z	20	36.1972	19.30667	−1.7843	123.0257

**Fig. 5:**
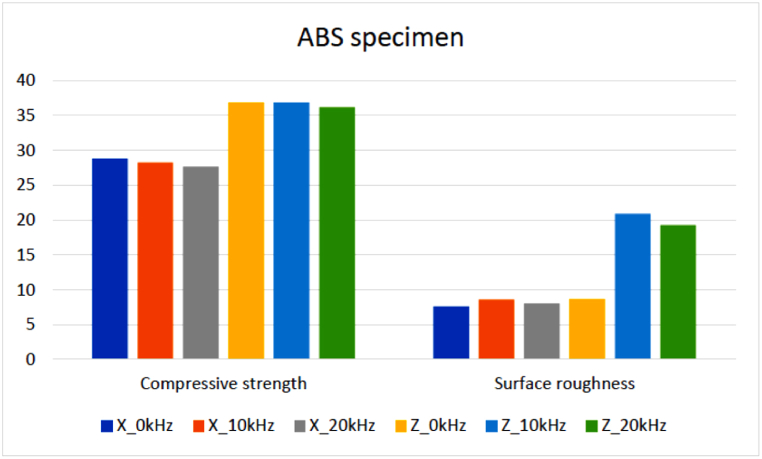
The compressive strength and surface roughness in different build orientations and ultrasonic frequency.

In contrast, in Z orientation, the surface roughness is increased by about 141.663% at 10 kHz and 123.026% at 20 kHz compared to 0 kHz. Based on the result, the ultrasonic vibration does not affect the compressive strength in X and Z orientation, as the values are very close to 0 kHz after the ultrasonic vibration is applied. However, the build orientation obviously affects the compressive strength as the compressive strength of samples built with Z-axis orientation is higher than with X-axis orientation. Furthermore, the build orientation and ultrasonic vibration have affected the surface roughness of ABS as the values are increased at 10 kHz and then decreased at 20 kHz, especially the effect that is obvious in Z-axis orientation. Fig. 5 shows the compressive strength and surface roughness in different build orientations and ultrasonic frequencies.

Table 4 shows PLA samples' average compressive strength and surface roughness results. Based on the result, the compressive strength built with Z-axis orientation is higher than those built with X-axis orientation, where the highest value in X-axis orientation is 28.8274 MPa at 0 kHz, and Z-axis orientation is 36.8588 MPa at 10 kHz. In the X-axis orientation, the compressive strength decreases when the ultrasonic vibration frequency increases. However, in Z-axis orientation, the compressive strength is increased by about 0.0109% at 10 kHz while decreasing by about 1.7843% at 20 kHz compared to 0 kHz. For the result of surface roughness for ABS samples, the samples built with X-axis orientation have a lower value than the Z-axis orientation. When the ultrasonic vibration was increased, the surface roughness of the samples built with X-axis orientation increased by about 12.9432% at 10 kHz and 5.7285% at 20 kHz. In contrast, in Z-axis orientation, the surface roughness increased by about 141.663% at 10 kHz and 123.026% at 20 kHz compared to 0 kHz.

**Table 4:** Result of compressive strength and surface roughness for PLA printed samples and its improvement using ultrasonic-assisted FDM.

Run	Control Factors		Response Values		Improvement compared to 0 kHz (%)	
	Build Orientation	Ultrasonic Frequency (kHz)	Compressive Strength (MPa)	Surface Roughness, Ra (μm)	Compressive Strength (MPa)	Surface Roughness, Ra (μm)
1	X	0	31.8556	7.2367	–	–
2	X	10	31.2301	12.1133	−1.9635	67.3871
3	X	20	32.293	10.9433	1.3731	51.2195
4	Z	0	36.5582	8.43	–	–
5	Z	10	36.4505	9.4067	−0.2946	11.5860
6	Z	20	36.2703	9.0267	−0.7875	7.0783

**Fig. 6:**
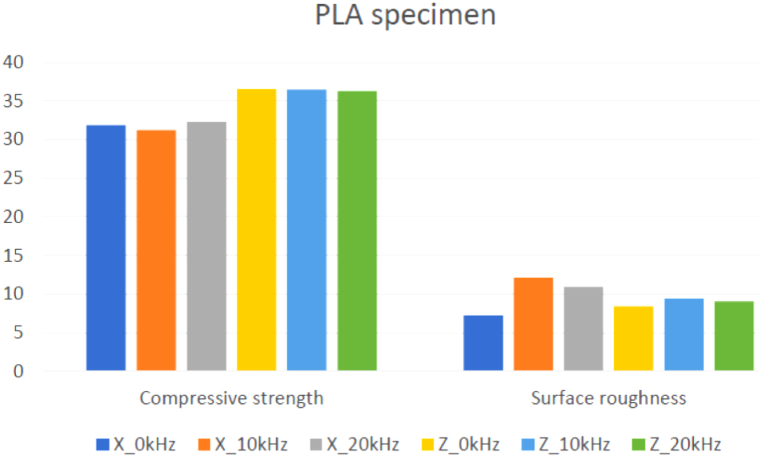
The results of compressive strength and surface roughness for PLA samples between different build orientations and ultrasonic frequency.

In contrast, the ultrasonic vibration does not affect the compressive strength in X and Z-axis orientation, as the values are very close to 0 kHz when the ultrasonic vibration is applied. However, the build orientation has an obvious effect on the compressive strength as the compressive strength of samples that are built with Z-axis orientation is higher than with X-axis orientation. Furthermore, the build orientation and ultrasonic vibration frequency have affected the surface roughness of ABS as the values are increased at 10 kHz and then decreased at 20 kHz, especially the effect that is obvious in Z-axis orientation. Fig. 6 shows the results of compressive strength and surface roughness for PLA samples between different build orientations and ultrasonic frequencies.

### 3.2 Microstructure analysis

**Fig. 7:**
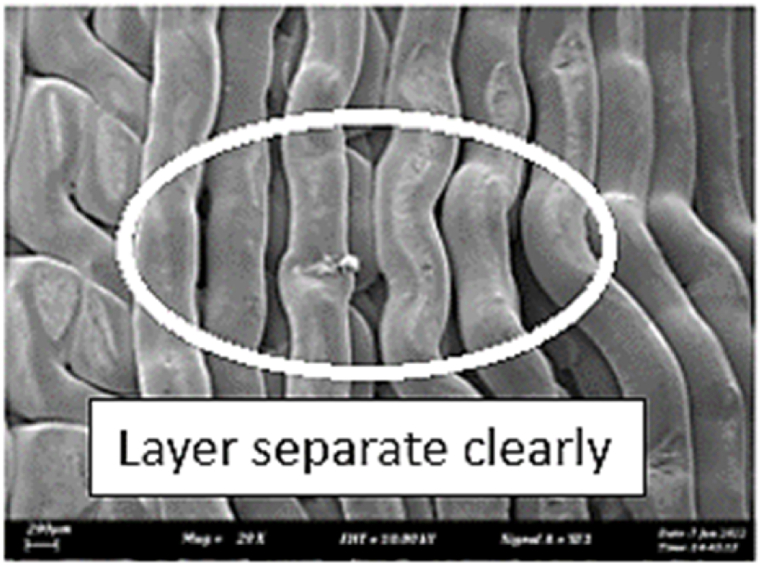
Microstructure inspection on ABS samples in X orientation with 0 kHz.

**Fig. 8:**
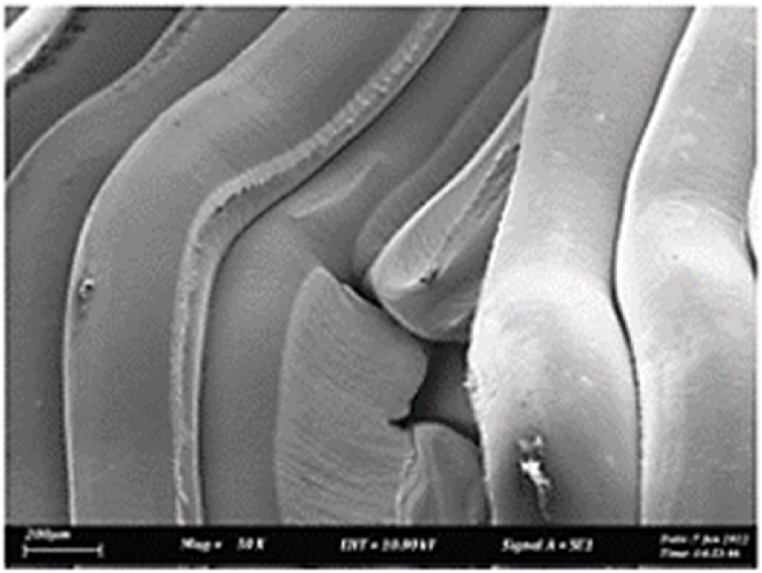
Microstructure inspection on ABS samples in X orientation with 10 kHz.

**Fig. 9:**
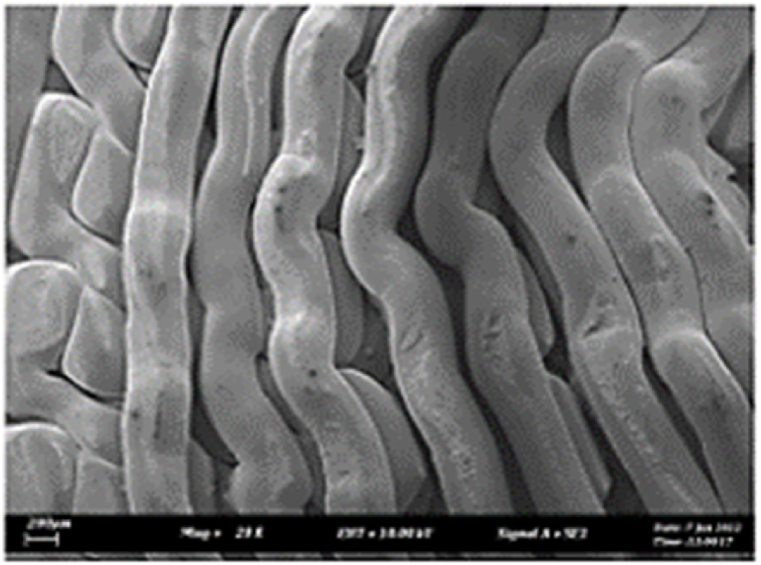
Microstructure inspection on ABS samples in X orientation with 20 kHz.

**Fig. 10:**
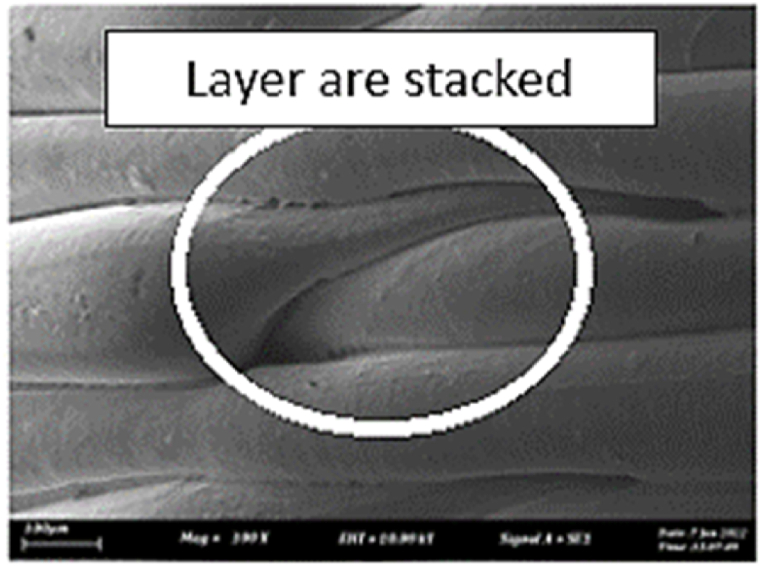
Microstructure inspection on ABS samples in Z orientation with 0 kHz.

As shown in Fig. 7, the samples printed with X-axis orientation and 0 kHz have a low bonding between layers as the layers are separated easily after the samples are compressed. Compared to Fig. 8, Fig. 9, which respect to the 10 kHz and 20 kHz, there is not much difference observed in microstructure, and this proves that the ultrasonic vibration is not significant to the compressive strength in X-axis orientation as the value of compressive strength does not have much different and very closed to each other. In Fig. 10, the microstructure of samples that were built with Z-axis orientation and 0 kHz shows that the layers are compressed and stacked, which results from the perpendicular direction of layers to the compressive load and leads to a higher compressive strength compared to X-axis orientation [42].

**Fig. 11:**
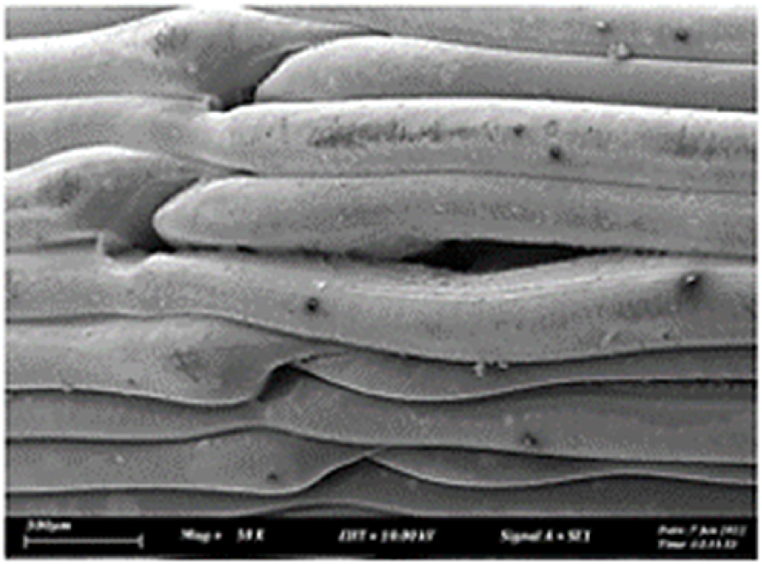
Microstructure inspection on ABS samples in Z orientation with 10 kHz.

**Fig. 12:**
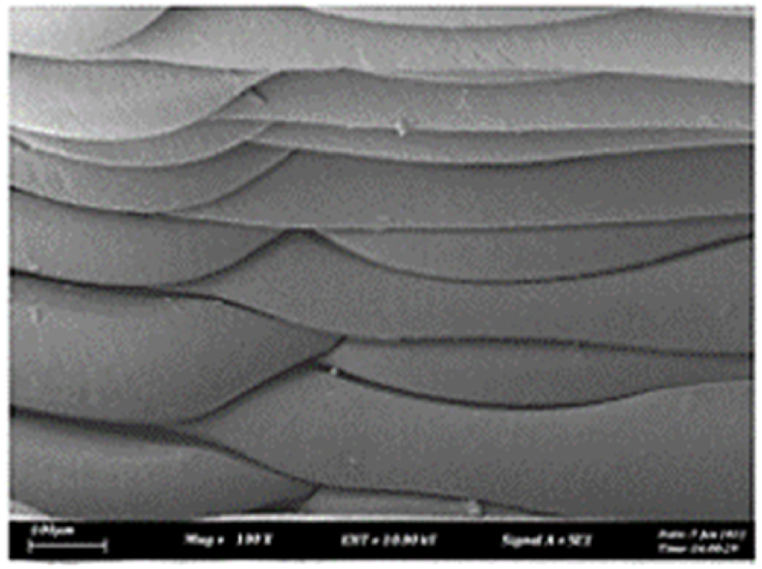
Microstructure inspection on ABS samples in Z orientation with 20 kHz.

**Fig. 13:**
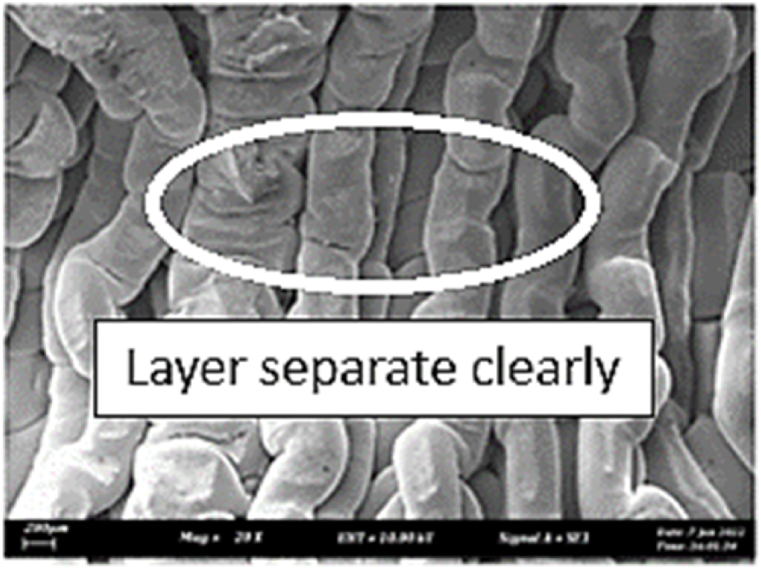
Microstructure inspection on PLA samples in X orientation with 0 kHz.

**Fig. 14:**
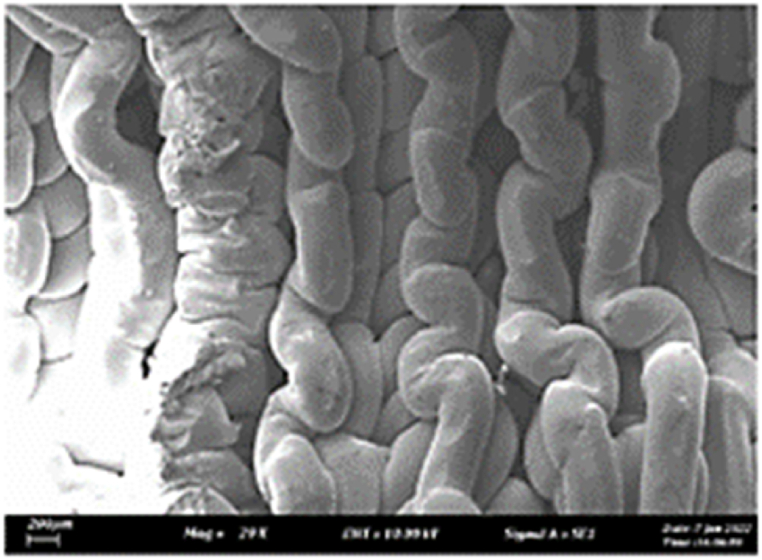
Microstructure inspection on PLA samples in X orientation with 10 kHz.

**Fig. 15:**
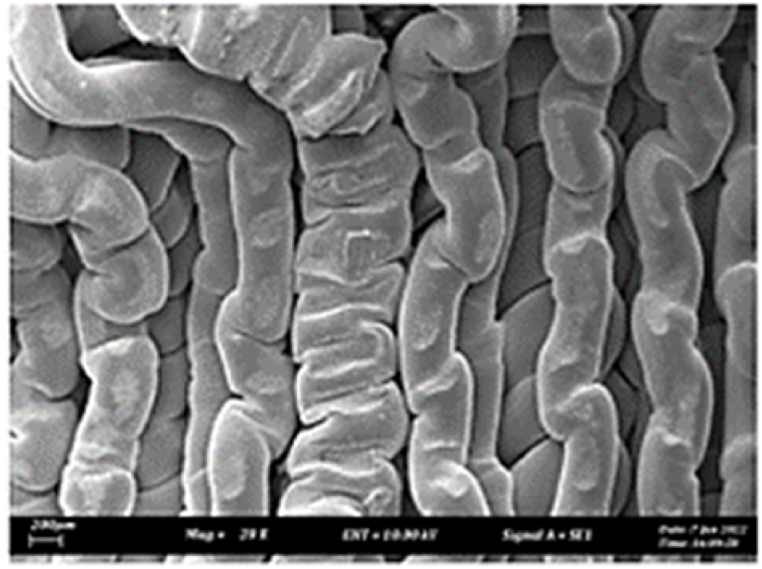
Microstructure inspection on PLA samples in X orientation with 20 kHz.

**Fig. 16:**
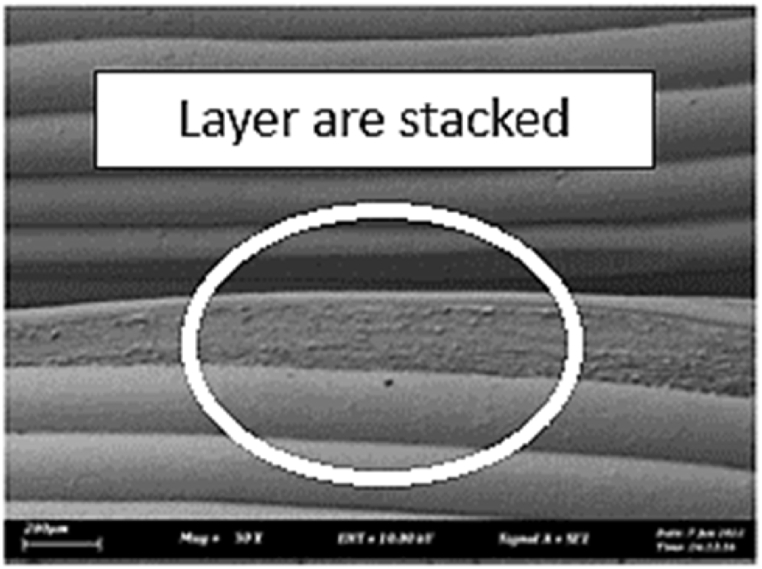
Microstructure inspection on PLA samples in Z orientation with 0 kHz.

**Fig. 17:**
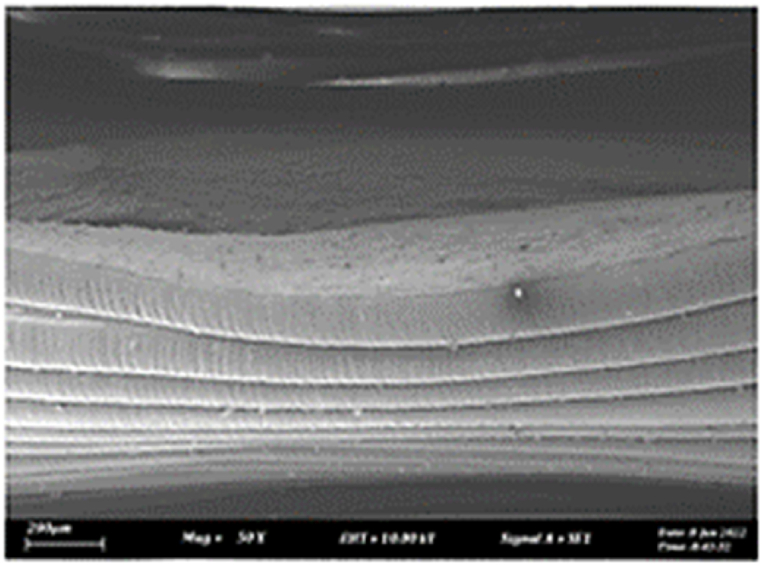
Microstructure inspection on PLA samples in Z orientation with 10 kHz.

**Fig. 18:**
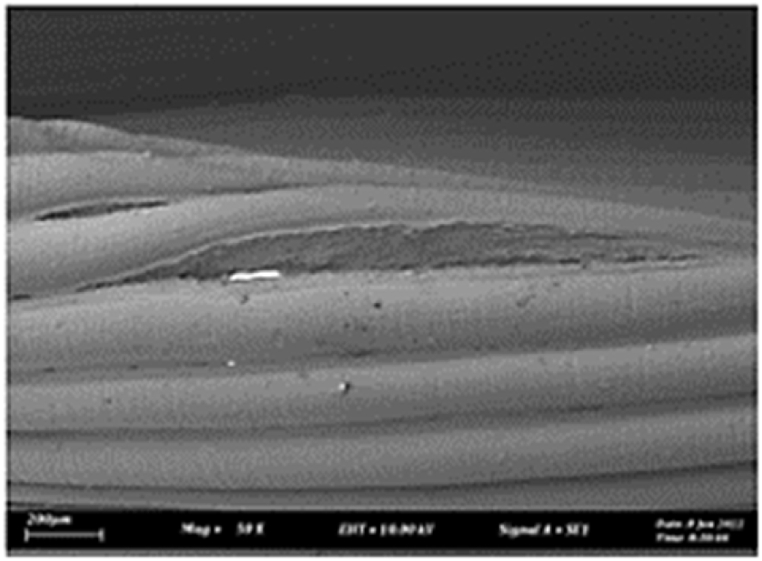
Microstructure inspection on PLA samples in Z orientation with 20 kHz.

However, in Fig. 11, Fig. 12, the samples built with Z-axis orientation in 10 kHz and 20 kHz, both microstructures do not show much difference compared to the bonding between layers in 0 kHz. Therefore, the compressive strength at 10 kHz and 20 kHz is similar to the value at 0 kHz. In the microstructure inspection of the PLA sample, the result is similar to the ABS samples, as the ultrasonic vibration does not bring much difference in the bonding between layers. Still, the orientation has caused a significant effect due to the perpendicular direction in layers to the compressive load. Fig. 13, Fig. 14, Fig. 15, Fig. 16, Fig. 17, Fig. 18 refer to the microstructure of PLA samples that were built with X and Z-axis orientation in 0 kHz, 10 kHz and 20 kHz after the compression testing.

### 3.3 ANOVA analysis

In the experiment, 18 readings were collected for each type of material and used to carry out the significant process parameters to the responses: compressive strength and surface roughness. The process parameters used in this study are build orientation and ultrasonic vibration, with two levels in build orientation and three levels in ultrasonic vibration. Based on the ANOVA analysis of the compressive strength of the ABS sample, as shown in Table 5, the build orientation shows that they are significant to the compressive strength as their P-value is less than 0.05. At the same time, the ultrasonic vibration and the two-way interactions are less significant to the compressive strength due to the higher value in P-value compared to 0.05. Furthermore, the compressive strength analysis had 96.52% in R-sq, 95.07% in R-sq (adjusted), and 92.16% in R-sq (predicted). As the value of R-sq is close to 100%, it had a strong relationship between the experiment results and the prediction results for the compressive strength analysis.

**Table 5:** ANOVA analysis of compressive strength of ABS samples.

Source	DF	Adj SS	Adj MS	*F*	*P*	Remarks
Build Orientation	1	323.692	323.692	329.53	0	Significant
Ultrasonic Frequency	2	2.414	1.207	1.23	0.327	Not Significant
2-way Interaction	2	0.586	0.293	0.3	0.747	Not Significant
Error	12	11.787	0.982			
Total	17	338.479	S = 0.991098	R^2^ = 96.52%	R^2^_adj_ = 95.07%	R^2^_pred_ = 92.16%

From the surface roughness analysis of ABS samples shown in Table 6, the build orientation, ultrasonic vibration frequency, and two-way interaction have a lower P-value below 0.05 which means they have a significant effect on the surface roughness. Besides, it had a value of 82.33% in R-sq, 74.97% in R-sq (adjusted), and 60.25% in R-sq (predicted). As the value of R-sq is still high enough, it had a strong relationship between the result of the experiments and the prediction for the surface roughness analysis.

**Table 6:** ANOVA analysis of surface roughness of ABS samples.

Source	DF	Adj SS	Adj MS	*F*	*P*	Remarks
Build Orientation	1	302.3	302.33	29.65	0.000	Significant
Ultrasonic Frequency	2	151.6	75.79	7.43	0.008	Significant
2-way Interaction	2	116.3	58.15	5.7	0.018	Significant
Error	12	122.4	10.2			
Total	17	692.6	S = 3.19321	R^2^ = 82.33%	R^2^_adj_ = 74.97%	R^2^_pred_ = 60.25%

In the ANOVA analysis of compressive strength for PLA samples shown in Table 7, the build orientation has a lower P-value below 0.05, meaning they have a significant effect on the surface roughness. At the same time, the ultrasonic vibration and two-way interaction have a higher P-value than 0.05, which means they are less significant to the compressive strength. Furthermore, it had a value of 95.97% in R-sq, 94.29% in R-sq (adjusted), and 90.93% in R-sq (predicted). The value of R-sq is close to 100%. It means that it had a strong relationship between the experiment results and the predicted results for the compressive strength analysis.

**Table 7:** ANOVA analysis of compressive strength of PLA samples.

Source	DF	Adj SS	Adj MS	*F*	*P*	Remarks
Build Orientation	1	96.61	96.6101	280.2	0	Significant
Ultrasonic Frequency	2	0.67	0.3348	0.97	0.407	Not Significant
2-way Interaction	2	1.17	0.585	1.7	0.224	Not Significant
Error	12	4.138	0.3448			
Total	17	102.587	S = 0.58719	R^2^ = 95.97%	R^2^_adj_ = 94.29%	R^2^_pred_ = 90.93%

For the ANOVA analysis of the surface roughness of PLA samples, as shown in Table 8, the ultrasonic vibration significantly affects the surface roughness as their P-values are lower than 0.05. For the orientation and two-way interaction, they are less significant to the surface roughness as their P-values are higher than 0.05. The surface roughness analysis of PLA samples had a value of 68.03% in R-sq, 54.71% in R-sq (adjusted), and 28.07% in R-sq (predicted). The value of R-sq is lower than 70%, which means a weak relationship exists between the experiment result and the prediction result for surface roughness analysis.

**Table 8:** ANOVA analysis of surface roughness of PLA samples.

Source	DF	Adj SS	Adj MS	*F*	*P*	Remarks
Build Orientation	1	5.882	5.882	3.25	0.097	Not Significant
Ultrasonic Frequency	2	27.591	13.796	7.62	0.007	Significant
2-way Interaction	2	12.753	6.377	3.52	0.063	Not Significant
Error	12	21.725	1.81			
Total	17	67.951	S = 1.3455	R^2^ = 68.03%	R^2^_adj_ = 54.71%	R^2^_pred_ = 28.07%

### 3.4 Main effect plot

**Fig. 19:**
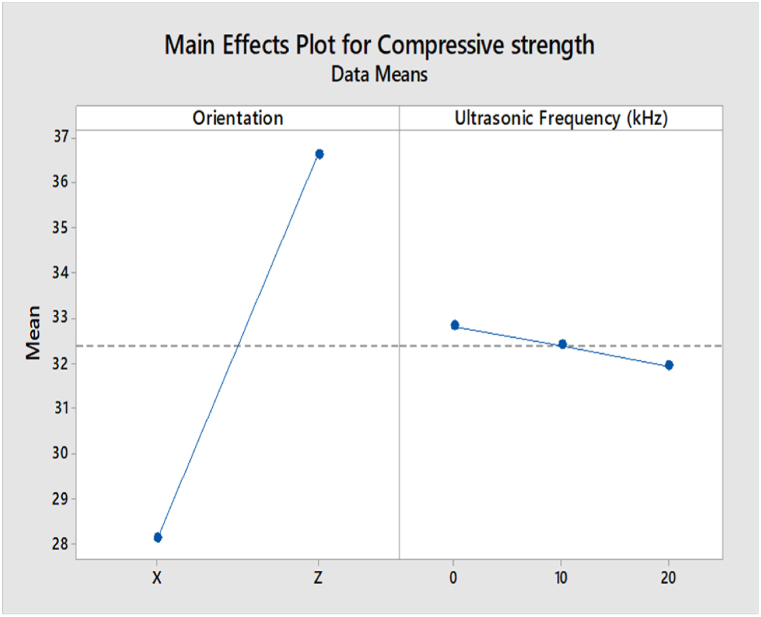
Main effect plot of compressive strength for ABS samples.

**Fig. 20:**
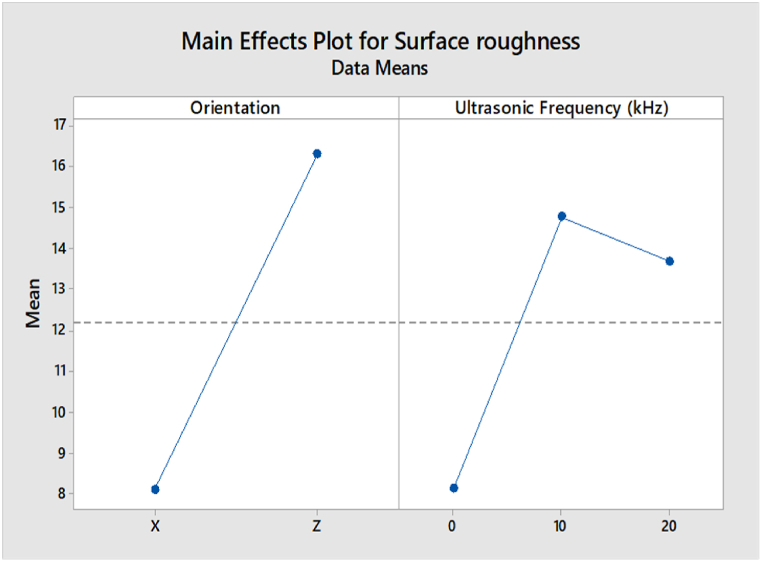
Main effect plot of surface roughness for ABS samples.

The main effect analysis refers to the effect of input parameters, namely build orientation and ultrasonic vibration, on the compressive strength and surface roughness. From Fig. 19, the compressive strength value in the Z-axis orientation is higher than in the X-axis orientation. This is because the layers in Z orientation have a perpendicular direction to the compressive load, leading to a higher compressive strength. In the ultrasonic vibration, the compressive strength is slightly decreasing when the ultrasonic vibration frequency increases. However, the value of compressive strength in 0 kHz, 10 kHz, and 20 kHz are very close to each other. Based on the main effect plot of surface roughness for ABS samples shown in Fig. 20, the value of surface roughness in the X-axis orientation is lower than in the Z-axis orientation. In the region of ultrasonic vibration, the surface roughness is increased drastically from 0 kHz to 10 kHz and then decreases from 10 kHz to 20 kHz.

**Fig. 21:**
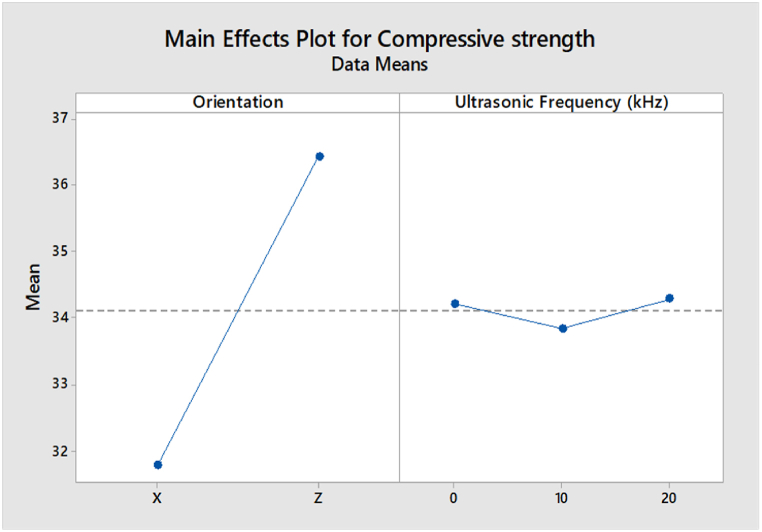
Main effect plot of compressive strength for PLA samples.

The main effect plot of compressive strength for PLA samples shown in Fig. 21 is similar to the ABS samples, where the value of compressive strength in Z-axis orientation is higher than the value in X-axis orientation due to the perpendicular direction of layers in Z orientation to the compressive load. In terms of ultrasonic vibration, there are slight differences between ABS and PLA samples, as the compressive strength is increased when the ultrasonic vibration is decreased to 10 kHz. Still, the value of compressive strength is then increased at 20 kHz. However, the value of compressive between 0 kHz, 10 kHz, and 20 kHz are very close to each other.

**Fig. 22:**
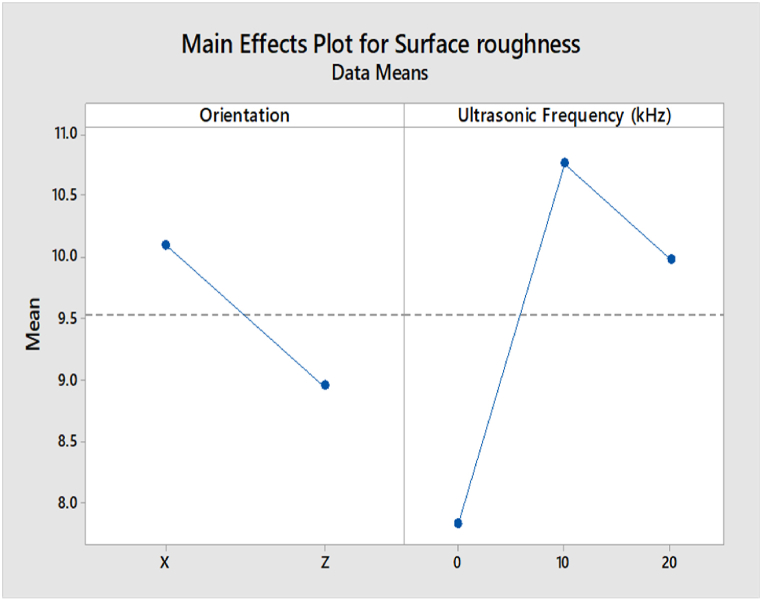
Main effect plot of surface roughness for PLA samples.

Based on Fig. 22, the main effect plot of surface roughness for PLA samples shows that the value of surface roughness in Z orientation is lower than in the X-axis orientation, which is different from the ABS samples. In ultrasonic vibration, the results are similar to the ABS samples, where the surface roughness is increased dramatically from 0 kHz to 10 kHz and then dropped from 10 kHz to 20 kHz.

### 3.5 Interaction plot

**Fig. 23:**
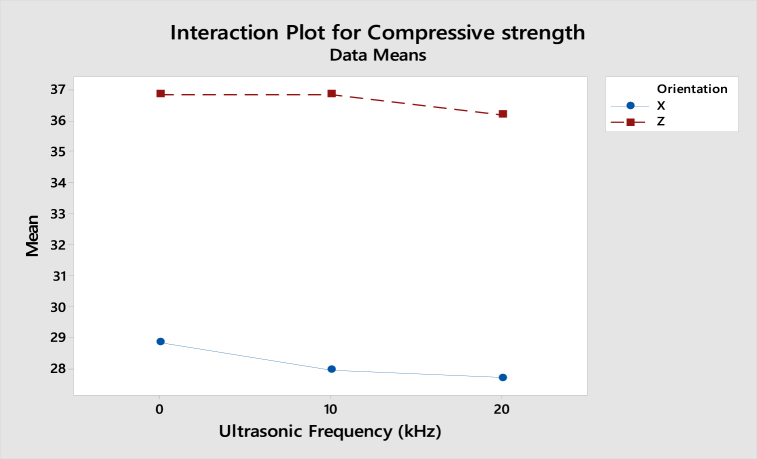
Interaction plot for compressive strength of ABS samples.

**Fig. 24:**
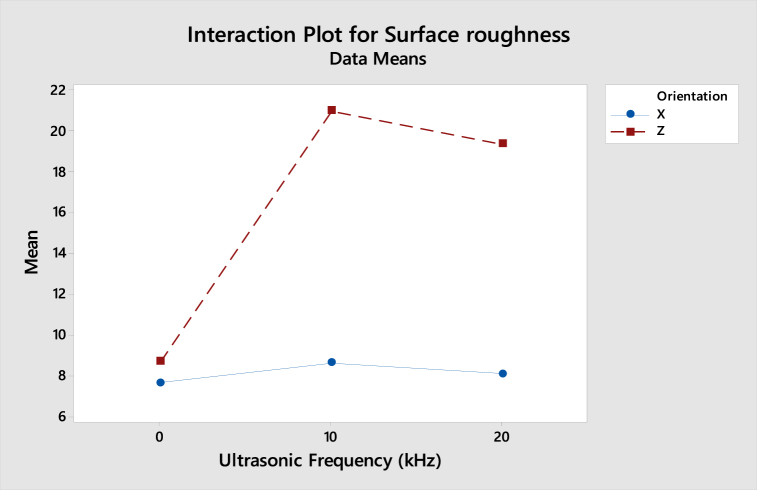
Interaction plot for surface roughness of ABS samples.

As shown in Fig. 23, the interaction plot of compressive strength for ABS samples shows that the gradient in X-axis and Z-axis orientation is slightly dropped from 0 kHz to 20 kHz. However, as the lines are parallel, there is no interaction effect between the build orientation and ultrasonic vibration to the compressive strength. For the interaction plot of surface roughness shown in Fig. 24, the value increased from 0 kHz to 10 kHz and then decreased from 10 kHz to 20 kHz. As the lines are still parallel, there is also no interaction effect between build orientation and ultrasonic vibration to the surface roughness for ABS samples.

**Fig. 25:**
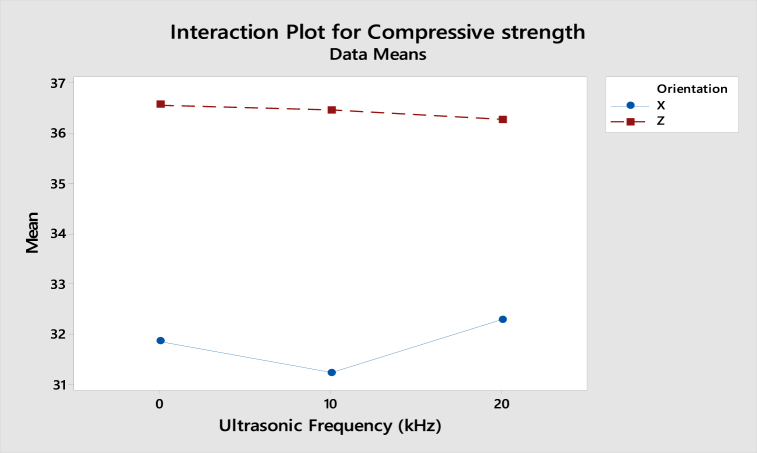
Interaction plot for compressive strength of PLA samples.

**Fig. 26:**
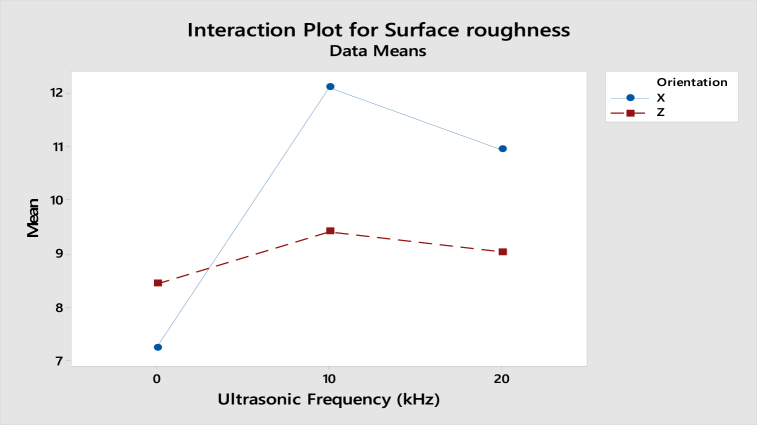
Interaction plot for surface roughness of PLA samples.

From the interaction plot of compressive strength for PLA samples shown in Fig. 25, there is a slight difference between X and Z-axis orientations, where the value of compressive strength in the Z-axis orientation decreases as the ultrasonic vibration increases. Still, the compressive strength in X-axis orientation is reduced from 0 kHz to 10 kHz and then increased from 10 kHz to 20 kHz. This shows a small interaction effect between the orientation and ultrasonic vibration to the compressive strength. In Fig. 26, the interaction plot of surface roughness for PLA samples, a cross-interaction effect occurred from 0 kHz to 10 kHz between the X and Z-axis orientation. The surface roughness of both orientations decreased from 10 kHz to 20 kHz.

### 3.6 Optimization plot

**Fig. 27:**
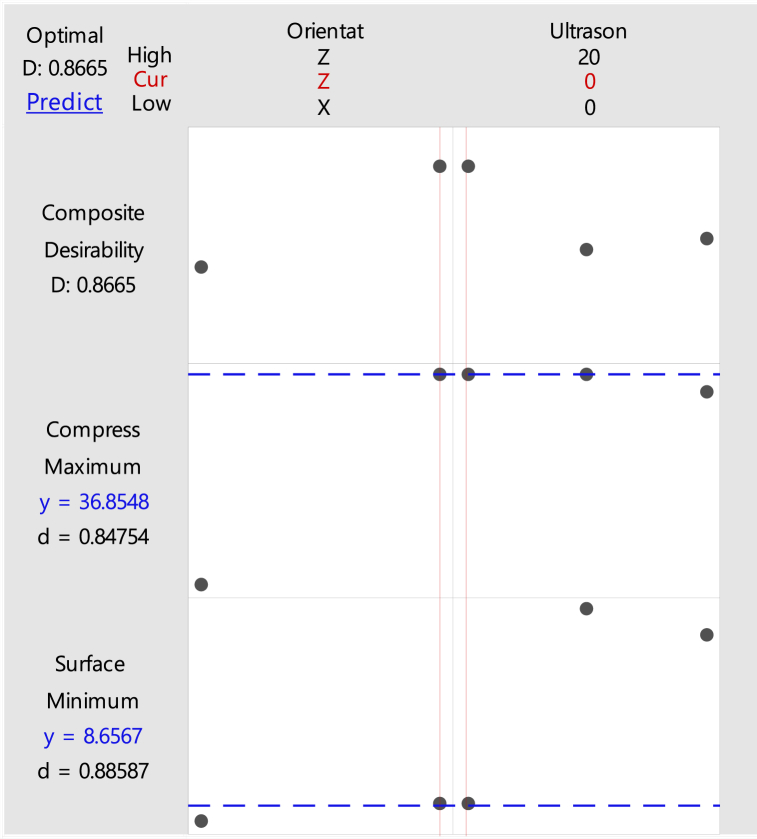
Optimization plot of ABS samples.

**Fig. 28:**
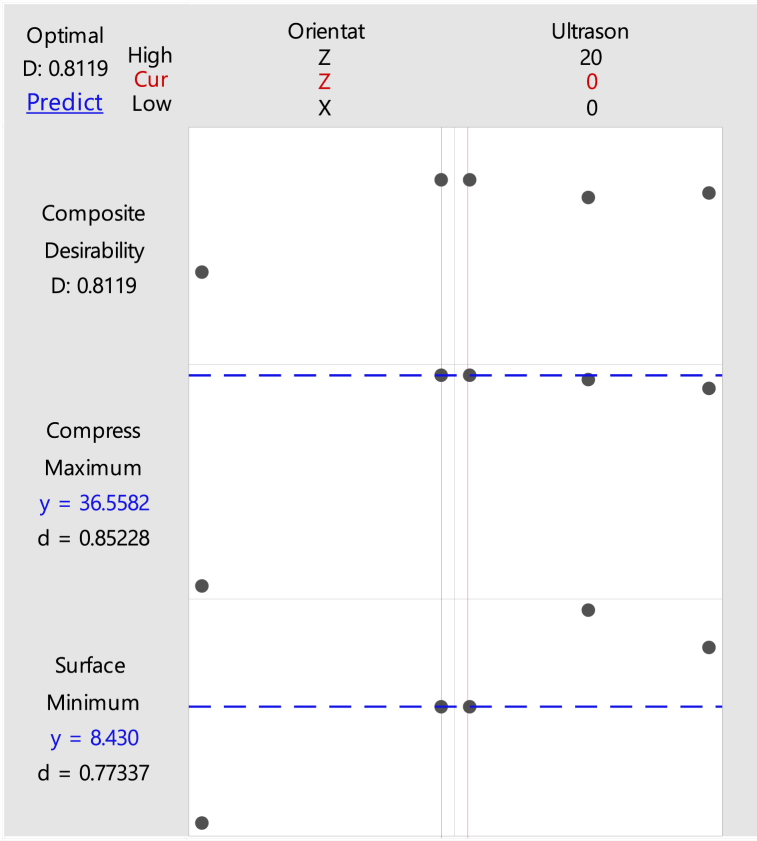
Optimization plot of PLA samples.

A high compressive and low surface roughness are required to have the best properties for the product. Therefore, the optimization parameters are performed with these settings where the compressive strength is maximized and the surface roughness is minimized. These settings are used to analyze the optimized parameters that have the highest compressive strength and the lowest surface roughness. By using Minitab software, the optimization in orientation and ultrasonic vibration for ABS and PLA samples are produced. From the result generated in Fig. 27, Fig. 28, the optimized parameters for ABS and PLA samples with the highest compressive strength and lowest surface roughness are the same: Z-axis in build orientation and 0 kHz in ultrasonic vibration frequency.

## 4 Conclusion

In conclusion, applying ultrasonic vibration on the open-source FDM printer in the printing process is feasible. The samples are analyzed through the data obtained from compressive testing, surface roughness testing and microstructure inspection. From the result obtained, the ultrasonic vibration does not have much effect on the compressive strength in both ABS and PLA materials but significantly affects the surface roughness for both materials as the surface roughness has decreased after applying the ultrasonic vibration during the printing process. The process parameter, namely build orientations, has a significant effect on the compressive strength of both materials and a substantial impact on the surface roughness in ABS material but less significant in PLA materials. High compressive and low surface roughness are required to have the best properties for the printed sample. Therefore, the optimization parameters are performed with these settings where the compressive strength is maximized and the surface roughness is minimized. The optimized parameters are found printing with Z-axis orientation and 0 kHz that shows the best result for the compressive strength and surface roughness for both ABS and PLA materials.

## Author contribution statement

Shajahan Maidin: Conceived and designed the experiments; Analyzed and interpreted the data.

Norilani Md. Nor Hayati: Performed the experiments.

Yap Yeong Sheng: Performed the experiments; Wrote the paper.

Ahmad Hilmi Muhammad: Contributed reagents, materials, analysis tools or data.

Thavinnesh Kumar Rajendran: Analyzed and interpreted the data; Contributed reagents, materials, analysis tools or data.

Shafinaz Ismail: Analyzed and interpreted the data; Wrote the paper.

## Funding statement

This work was supported by the Universiti Teknikal Malaysia Melaka (UTeM) and the Ministry of Higher Education Malaysia for awarding the Fundamental Research Grant Scheme (FRGS) grant number FRGS/1/2021/TK0/UTEM/02/24.

## Data availability statement

Data will be made available on request.

## Declaration of competing interest

The authors declare that they have no known competing financial interests or personal relationships that could have appeared to influence the work reported in this paper.
